# Retrospective Comparative Study of the Effects of Dendritic Cell Vaccine and Cytokine-Induced Killer Cell Immunotherapy with that of Chemotherapy Alone and in Combination for Colorectal Cancer

**DOI:** 10.1155/2014/214727

**Published:** 2014-08-18

**Authors:** Jingxiu Niu, Yanjie Ren, Tianyu Zhang, Xuejing Yang, Wei Zhu, Hui Zhu, Jing Li, Jiali Li, Yan Pang

**Affiliations:** ^1^Department of Oncology, Tianjin Union Medicine Center, No. 190 Jieyuan Road, Hongqiao District, Tianjin 300121, China; ^2^Shanghai Claison Biotechnology Co. Ltd., Shanghai, China

## Abstract

*Purpose.* This retrospective study determined the delayed-type hypersensitivity (DTH) skin test and safety of dendritic cell (DC) vaccine and cytokine-induced killer (CIK) cell immunotherapy and the survival compared to chemotherapy in 239 colorectal cancer (CRC) patients. *Methods.* DTH and safety of the immunotherapy were recorded. The overall survival (OS) and disease free survival curves were compared according to the immunotherapy and/or chemotherapy received with Kaplan-Meier estimates. *Results.* Of the 70 patients who received immunotherapy, 62.86% had a positive DTH skin test, 38.57% developed fever, 47.14% developed insomnia, 38.57% developed anorexia, 4.29% developed joint soreness, and 11.43% developed skin rash. For 204 resectable CRC patients, median survival time (MST) (198.00 days) was significantly longer in patients with immunotherapy plus chemotherapy than with chemotherapy alone (106.00 days) (*P* = 0.02). For 35 patients with unresectable or postsurgery relapsed CRC and who were confirmed to be dead, no statistical difference was observed in the MST between the patients treated with immunotherapy and with chemotherapy (*P* = 0.41). MST in the patients treated with chemotherapy plus immunotherapy was 154 days longer than that of patients treated with chemotherapy alone (*P* = 0.41). *Conclusions.* DC vaccination and CIK immunotherapy did not cause severe adverse effects, induce immune response against CRC, and prolong OS.

## 1. Introduction

Oxaliplatin and 5-FU-based chemotherapy are the standard treatment modalities for high-risk stage II, stage III, and stage IV CRC patients [1, 2]. Regimens of capecitabine, CapeOX, and FOLFOX 4 are also commonly used in clinical treatment, and they result in survival benefits. Oxaliplatin and/or 5-FU can kill both rapidly dividing CRC cells and normal cells [3, 4]. As a result, hair loss, diarrhea, nausea, and vomiting are commonly observed in CRC patients undergoing chemotherapy treatment. In addition to these adverse effects, severe adverse events including myelosuppression, immunosuppression, and permanent organ damage to the heart, lung, liver, and kidneys can occur during chemotherapy in CRC patients [5]. Because of their poor general condition, advanced CRC patients often cannot withstand the toxic effects of chemotherapy and, therefore, do not receive adequate therapy. In addition to its toxicity, chemotherapy sensitivity declines over time [6, 7]. CRC that recurs after an initial response to oxaliplatin and 5-FU tends to be resistant to subsequent chemotherapy with different drugs. Complete tumor eradication is rarely achieved in most advanced CRC patients treated with chemotherapy [7]. Because of the limited clinical benefit and toxicity of chemotherapy, immunotherapy may be a better option for improving OS in advanced CRC. Immunotherapy uses the body's immune system to attack cancer cells. Antigen-specific T cell dysfunction is common in cancer patients. As a result, tumor cells escape immune surveillance. Restoring the immune system may be a viable option to improve cancer treatment. Immunotherapy may be a promising and safe approach for cancer eradication [8, 9]. Unlike routine therapies, immunotherapy may induce an effective antitumor immune response without adverse effects. Immunotherapy with dendritic cell (DC) and cytokine-induced killer (CIK) cells is targeted to kill residual cancer cells, which are the main cause of cancer recurrence and metastasis. DC and CIK immunotherapy has been shown to be well tolerated with excellent compliance in cancer patients [10, 11].

The purpose of this study was to compare the therapeutic efficacy in terms of survival prolongation of DC cells and CIK cells immunotherapy and chemotherapy alone and in combination in advanced CRC patients.

## 2. Patients and Methods

### 2.1. Patients

CRC patients who were treated at the Department of Oncology, Tianjin Union Medicine Center, from February 1, 2012 to September 30, 2013 were included in the study. Inclusion criteria consist of: (1) patients with histologically or cytologically diagnosed CRC and adequate kidney liver, coagulation, and bone marrow function; (2) resectable (stage II and stage III) CRC patients who accepted primary tumor resection and postoperative adjuvant chemotherapy and had an elevated serum carcinoembryonic antigen (CEA) level before surgery and a normal serum CEA level (5 ng/mL) within 1 month after surgery; and (3) advanced CRC patients (relapsed or metastatic CRC after surgery and unresectable CRC) who were confirmed to be dead of any cause.

The resectable CRC patients were divided into 2 groups according to the treatment they received. Patients who received routine adjuvant postsurgery chemotherapy alone were defined as group S + C (control group). Patients who received DC and CIK cells immunotherapy plus chemotherapy within 6 months after surgery were defined as group S + C + I (immunotherapy group). Advanced CRC patients were divided into 3 groups according to the treatment modalities they received: group I, DC cells and CIK cells immunotherapy; group C, chemotherapy; and group I + C, DC vaccine and CIK cell immunotherapy plus chemotherapy. The interval between immunotherapy and chemotherapy had to be less than 3 months; it is to ensure that the result of clinical efficacy is the result of immunotherapy and chemotherapy function together rather than unilateral.

### 2.2. Design of DC Cells and CIK Cells Therapy

The schedule of DC and CIK therapy was performed in accordance with the “Treatment with Autologous Immune Cells (T cells, NK cells)” class III medical techniques policy of the Ministry of Health of China. This study protocol was approved by ethical committees of the hospital. Written informed consent was obtained from each patient before treatment initiation. Adequate renal and coagulation function and peripheral lymphocyte and monocyte numbers greater than 1 × 10^9^/L were required for patients to receive DC cells and CIK cells therapy.

### 2.3. Collection of Peripheral Blood Mononuclear Cells

On day 0, peripheral blood mononuclear cells (PBMCs) were collected by leukapheresis using a Fresenius KABI System (Germany) with an electrocardiogram monitoring system. PBMCs were cultured overnight and adherent cells (monocytes) and nonadherent cells (lymphocytes) were separated.

### 2.4. Preparation of DCs and CIK Cells

Tumor lysate was first prepared for pulsing DCs. A single cell suspension of SW480 colon cancer cell line was dissociated by ultrasound and centrifugation at 600 ×g for 30 min, and the supernatants were collected as tumor lysate. DCs were obtained by culturing the adherent cells through stimulating with tumor lysate, granulocyte macrophage colony-stimulating factor, interleukin (IL)-4, and tumor necrosis factor for 7 days [11, 12]. For preparation of CIK cells, SW480 cells CIK cells were obtained by culturing the nonadherent cells through stimulating with interferon *γ*, CD3 monoclonal antibody, and IL-2 for 10 days.

### 2.5. Quality Control of DCs and CIK Cells

The immune phenotypes of HLA2DR, CD80, and CD83 for DCs and CD3, CD8, and CD56 for CIK were analyzed by flow cytometry [13]. The bacteria, fungus, and endotoxin levels in cultured DC and CIK samples met the release criteria for infusion [10, 14, 15].

### 2.6. Schedule of DC Cells and CIK Cells Infusion

DCs (1 × 10^7^) in 100 mL 0.9% normal saline (NS) were intravenously infused on days 8, 15, and 22 and injected intradermally on days 29, 36, and 43. CIK cells (1 × 10^9^) in 100 mL NS were intravenously infused on days 11, 12, 13, and 14. An interval of 4–6 hours was needed to receive immunotherapy and chemotherapy within the same day [15].

### 2.7. Delayed-Type Hypersensitivity Testing of DC Vaccine and CIK Cell Infusion

For delayed-type hypersensitivity (DTH) tests, 4 *μ*g of tumor lysate was intradermally injected 1 week after the last DC infusion and results were examined 48 hours later. An induration greater than 2 mm in diameter around the injection site was considered as a positive DTH response (Table 2).

### 2.8. Safety Evaluation of DC Cells and CIK Cells Immunotherapy

Adverse effects such as fever, insomnia, anorexia, joint soreness, and skin rash were evaluated during DC cells and CIK cells infusion. Several adverse effects occurred simultaneously in the same patient (Table 2).

### 2.9. Chemotherapy Schedules

Capecitabine was administered orally twice daily at a dose of 1000 mg/m^2^ on day 1 to day 14. This regimen was repeated every 3 weeks [16, 17]. For the capecitabine plus oxaliplatin (CapeOX) regimen, oxaliplatin was intravenously infused at a dose of 130 mg/m^2^ on day 1, and capecitabine was administered orally twice daily at a dose of 1000 mg/m^2^ on day 1 to day 14. The regimen was repeated every 3 weeks. The FOLFOX 4 (oxaliplatin, leucovorin [LV], and 5-FU) regimen consisted of intravenous oxaliplatin infusion (85 mg/m^2^) on day 1, intravenous LV infusion (200 mg/m^2^) on day 1 and day 2, and intravenous bolus injection (400 mg/m^2^) and 22 h infusion (600 mg/m^2^) of 5-FU on day 1 and day 2. This regimen was repeated every 2 weeks. To receive chemotherapy, patients were required to have adequate kidney, liver, and bone marrow function [17–20].

### 2.10. Follow-Up

Overall survival (OS) was recorded for the advanced CRC patients. For the resectable CRC patients, CEA levels were measured before operation and within 1 month after operation, and patients were followed up until November 14, 2013. The follow-up occurred 3 to 4 weeks postoperatively. Serum CEA levels were monitored every 3 weeks just before receiving adjuvant chemotherapy. Serum CEA levels were recorded every 3 months for the first 2 years of follow-up and thereafter every 6 months. Based on the restrictions presented below, the time from the date of surgery to the date of the first rise in serum CEA level above the normal upper limit reflected the duration of tumor-free status in patients, and, therefore, it was used to provide an estimate of disease-free survival (DFS). Patients with serum CEA levels more than 5 ng/mL before surgery, which was the reference cutoff in our hospital, were considered to have CEA-producing tumors, and, therefore, CEA could be used as an index for evaluation of tumor recurrence. Patients with serum CEA levels below 5 ng/mL within 1 month of surgery were considered as a radical surgery. Patients whose serum CEA level was above the normal upper limit 3 consecutive times during follow-up were considered to have tumor recurrence [21–24].

### 2.11. Data Collection and Statistical Analysis

The patients were followed up until November 14, 2013. In advanced CRC patients, OS was defined as the time from the date of study enrollment to the date of death from any cause. In resectable CRC patients, DFS was defined as the time from the date of the first rise in serum CEA level above the normal upper limit to the date of surgery.

Clinical data of the patients including diagnostic procedures and treatment were collected from the inpatients electronic medical records of our hospital and reanalyzed using EpiData database (version 3.02). Particular attention was paid to collecting data related to primary rumor resection, chemotherapy, and DC cells and CIK cells immunotherapy. Statistical analyses were carried out using the SPSS (version 19.0) statistical software package, which was docked with the EpiData database. DFS and OS curves were calculated using the Kaplan-Meier method. A *P* value less than 0.05 was considered statistically significant.

## 3. Results

### 3.1. Patient Characteristics

A total of 239 CRC patients (204 resectable CRC patients and 35 advanced CRC patients) were enrolled in the study during an 8-month period from February 1, 2012 to September 30, 2012 and followed up until November 14, 2013. Patient characteristics are shown in Table 1. Of the 239 patients, 133 male patients and 106 female patients were with a mean age of 64.2 ± 12.05 (range 28–86) years. The primary tumor was located in the colon and rectum in 92 patients and 147 patients, respectively. One patient, 188 patients, and 50 patients had well-differentiated, moderately differentiated, and poorly differentiated tumors, respectively. Of the unresectable CRC patients, 24 (20.8%) patients underwent primary tumor resection, 8 (8.3%) patients received radiotherapy, 28 patients received chemotherapy, and 24 patients received DC cells and CIK cells immunotherapy. Of the 28 patients who received chemotherapy, 5 patients received the capecitabine regimen, 6 patients received the CapeOX regimen, 12 patients received the FOLFOX 4 regimen, and 5 patients received other regimens.

Of the 204 resectable CRC patients, 85 patients had stage II disease and 119 patients had stage III disease. These 204 patients were divided into 2 groups according to the treatment they received. The control group (*n* = 130) received primary tumor resection followed by adjuvant chemotherapy. The immunotherapy group (*n* = 74) received primary tumor resection followed by adjuvant chemotherapy plus adjuvant DC cells and CIK cells immunotherapy. The 35 advanced CRC patients were divided into 3 groups according to the treatment they received: group C (*n* = 11), chemotherapy alone; group I (*n* = 7), DC cells and CIK cells immunotherapy alone; and group I + C (*n* = 17), DC cells and CIK cells immunotherapy plus chemotherapy.

### 3.2. Immune Response and Safety of DC Cells and CIK Cells Therapy

Immune response and safety of DC cells and CIK cells therapy were recorded in 70 of the 74 patients in group I. These parameters were not evaluated in 4 patients because of incomplete follow-up data. Of the 70 group I patients, 44 (62.86%) patients had a positive immune response based on the DTH skin test. Twenty-seven (38.57%) patients developed fever, 33 (47.14%) patients developed insomnia, 27 (38.57%) patients developed anorexia, 3 (4.29%) patients developed joint soreness, and 8 (11.43%) patients developed skin rash (Table 2). Severe adverse events were not observed.

### 3.3. Serum CEA Levels in Resectable CRC Patients

Mean CEA level was significantly lower after operation compared with before operation in the 204 resectable CRC patients (*P* = 0.81). Mean serum CEA level before and after surgery was 15.88 ± 20.63 (range 5–48) ng/mL and 2.78 ± 1.11 (range 5–48) ng/mL, respectively.

At the end of follow-up, serum CEA levels significantly rose in 48 of the 204 resectable CRC patients including 17 of the 74 (23.0%) immunotherapy group patients and 31 of the 130 (23.8%) control group patients (*P* = 0.58). The mean serum CEA level of these patients was 8.21 ± 5.96 (range 5–48) ng/mL. Presurgery and postsurgery CEA levels and tumor recurrence were similar between the immunotherapy and control groups (Table 3).

### 3.4. DFS Based on Serum CEA Level

**Figure 1:**
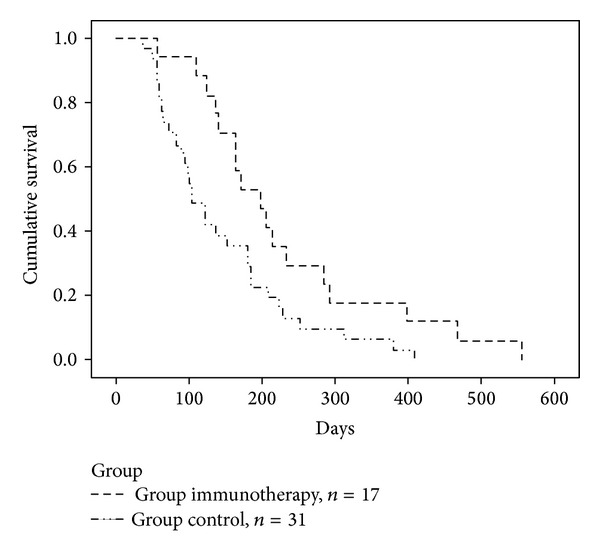
DFS curves by the Kaplan-Meier estimate in immunotherapy group and control group (*P* = 0.024).

The 204 resectable CRC patients were followed up for 489.2 ± 160.4 (range 441–652) days. At the end of follow-up, serum CEA levels were increased in 48 of the 204 patients including 17 patients in the immunotherapy group and 31 patients in the control group. Median survival time (MST) was significantly longer in the immunotherapy group than in the control group (*P* = 0.02) (Table 4). MST was prolonged for 92 days in the immunotherapy group compared with the control group (198 days versus 106 days). The DFS curves for the immunotherapy and control groups are shown in Figure 1.

### 3.5. Comparison of OS and MST between Chemotherapy and Immunotherapy in Advanced CRC Patients

**Figure 2:**
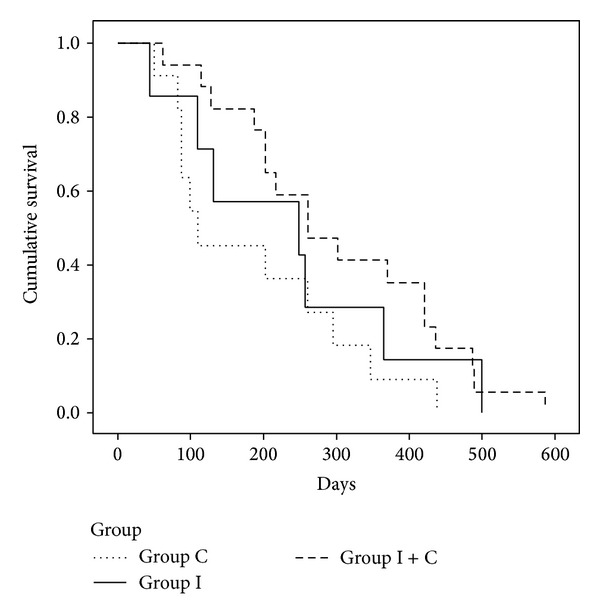
OS curves by the Kaplan-Meier estimate for patients with chemotherapy, immunotherapy alone, and immunotherapy plus chemotherapy of DC vaccine and CIK.

MSTs of the I, C, and I + C groups are shown in Table 5. MST was not significantly different between the I and C groups (249 days versus 110 days; *P* = 0.41). MST was significantly longer in the I + C group than in the C group (*P* = 0.04). MST was prolonged 154 days in the I + C group compared with the C group (264 versus 110 days). MST was not significantly different between the I and I + C groups (249 days versus 264 days; *P* = 0.47). The OS curves for the I, C, and I + C groups are shown in Figure 2.

## 4. Discussion

DTH as an indicator of immune response can serve as an efficacy end point for DC cells and CIK cells immunotherapy. OS is a powerful index to measure therapeutic efficacy in advanced CRC. Indices to evaluate therapeutic efficacy in early-stage CRC postsurgery are lacking. CEA is the most widely used tumor marker for the management of CRC [25, 26]. CEA is currently used to detect recurrent disease after curative resection and is believed to be effective indicator of postoperative mortality. Postoperative CEA level is an important indicator for both OS and DFS rates in CRC patients. CEA is an early marker for tumor recurrence. CEA level can detect tumor recurrence approximately 5 months earlier compared with clinical symptoms and imaging diagnosis. Quantitative measurement of serum CEA can be readily, easily, and inexpensively obtained. In this study, patients with serum CEA levels below 5 ng/mL before surgery were excluded because they were considered to have non-CEA-producing tumors and, therefore, CEA could not be used as an index for evaluation of tumor recurrence. The high false positive rate of CEA testing in CRC, which often results from incidental rises in CEA caused by benign gastrointestinal disorders, has been a main problem with its use. Serum CEA remains in the circulation for a period of time and requires several weeks to return to normal. It is believed that incidental rises in CEA can be filtered, and a higher specificity is expected by repetitive measurements of CEA at an interval of several weeks. In our study, a rise in the serum CEA level was defined as a CEA level above the normal upper limit for 3 consecutive follow-ups. Abnormal serum CEA levels in some patients persisted after surgery, which usually indicates the presence of residual microscopic disease. Patients with serum CEA levels above 5 ng/mL within 1 month after surgery were excluded from this study, because they were considered as nonradical surgery. If there is no residual disease after tumor resection, the serum CEA level should remain within normal range. If the CEA level rises, it is possible that a persistent source of CEA, such as a hidden metastasis or recurrent tumor is present. Based on these circumstances, the period from the date of surgery to that of the first rise in serum CEA level above the normal upper limit reflects the duration of tumor-free status, and this might provide another method to measure DFS [27, 28].

Based on the clinical observations of our study, 44 (62.86%) patients developed a positive immune response to DC cells and CIK cells immunotherapy based on the DTH skin test. Immunotherapy-related adverse effects including fever, insomnia, anorexia, joint soreness, and skin rash were observed. In general, these adverse effects were mild and resolved without the need for additional treatment. Severe adverse events were not observed. A rise in serum CEA level was detected in 17 of the 74 (23.0%) patients in the immunotherapy group and 31 of the 130 (23.8%) patients in the control group. Although the number of patients with an elevated CEA level during follow-up was not significantly different between the 2 groups, MST, defined as the time from the date of surgery to the date of the first rise in serum CEA level, was significantly longer (92 days) in the immunotherapy group than that in the control group. MST was not significantly different between patients who received immunotherapy and those who received chemotherapy. MST was 154 days longer in the patients who received chemotherapy plus immunotherapy than in patients who received chemotherapy alone. This indicates that DC vaccine and CIK cell immunotherapy and chemotherapy may have a similar effect on survival in advanced CRC patients. Combined immunotherapy and chemotherapy may have a synergistic effect on survival compared with chemotherapy alone.

DC cells and CIK cells immunotherapy can induce an immune response against CRC and prolong OS and DFS. The therapy was safe and no severe adverse effects were observed. DC cells and CIK cells immunotherapy and chemotherapy had a similar survival benefit in CRC patients. Combined immunotherapy and chemotherapy had a synergistic effect on survival compared with chemotherapy alone. Immunotherapy represents a viable treatment option to benefit CRC patients.

## 5. Conclusion

DC vaccination and CIK cell therapy were safe and no severe adverse effects were observed. DC cells and CIK cells therapy were able to stimulate the patients to use their own immune system against cancer. As a result, DFS and OS were prolonged. Immunotherapy and chemotherapy had the same effect on survival in CRC patients. Combined immunotherapy and chemotherapy may have a synergistic effect on survival compared with chemotherapy alone [29–31].

## Figures and Tables

**Table 1:** Characteristics of patients.

Characteristics	Number		
	Resectable	Advanced	Total
Number	204	35	239
Age (years)			
Range	28–83	40–86	28–86
Mean ± SD	61.74 ± 10.28	62.1 ± 8.10	64.2 ± 12.05
Gender			
Male	116	17	133
Female	88	18	106
Tumor location			
Colon	74	18	92
Rectum	130	17	147
Differentiation degrees			
High	1	0	1
Middle	161	27	188
Low	42	8	50
UICC stages			
I	0	0	0
II	85	0	85
III	119	0	119
IV + recurrence	0	35	35
Background of treatments			
Surgery	204	24	228
Radiology	0	8	8
Chemotherapy	204	28	232
DCs + CIK therapy	74	24	98

**Table 2:** Side effects in immunotherapy with DCs and CIK cells in immunotherapy group (*n* = 70).

Characteristics	Number of positives (%)	Number of negatives (%)
DTH	44 (62.86%)	26 (37.14%)
Fever	27 (38.57%)	43 (61.43%)
Insomnia	33 (47.14%)	37 (52.86%)
Anorexia	27 (38.57%)	43 (61.43%)
Joint soreness	3 (4.29%)	67 (95.71%)
Skin rash	8 (11.43%)	62 (88.57%)

**Table 3:** Serum CEA levels of presurgery, postsurgery, and tumor recurrence in both immunotherapy group and control group.

Group	Total	Immunotherapy group	Control group	*P* value
Presurgery				
Number	204	74	130	
Mean ± SD	15.88 ± 20.63	13.79 ± 11.99	17.05 ± 24.13	0.13
Range	10–48	10–48	10–48	
Postsurgery				
Number	204	74	130	
Mean ± SD	2.78 ± 1.11	2.91 ± 1.10	2.70 ± 1.12	0.81
Range	2–5	2–5	2–5	
Tumor recurrence				
Number/total number	48/204	17/74 (23.0%)	31/130 (23.8%)	0.89
Mean ± SD	8.21 ± 5.96	7.86 ± 6.05	8.40 ± 6.00	0.58
Range	5–10	5–10	5–10	

**Table 4:** Comparison of MST in immunotherapy group and control group.

Group	Number	MST (days)	ΔMST (days)	*χ* ^2^	(*P* value)
Immunotherapy	17	198.00	92.00	5.109	0.02
Control	31	106.00			

**Table 5:** Comparison of MST for patients with chemotherapy, immunotherapy alone, and immunotherapy plus chemotherapy of DC vaccine and CIK cells.

Group	Number	MST (day)	ΔMST	*χ* ^2^	(*P* value)
C versus I					
C	11	110	139	0.694	0.41
I	7	249			
C versus I + C					
C	11	110	154	4.127	0.04
I + C	17	264			
I versus I + C					
I	7	249	15	0.535	0.47
I + C	17	264			
